# Severe thoracic trauma – still an independent predictor for death in multiple injured patients?

**DOI:** 10.1186/s13049-017-0469-7

**Published:** 2018-01-08

**Authors:** Michael Grubmüller, Maximilian Kerschbaum, Eva Diepold, Katharina Angerpointner, Michael Nerlich, Antonio Ernstberger

**Affiliations:** Department of Trauma Surgery, University Medical Center Regensburg, Franz-Josef-Strauss-Allee 11, D-93053 Regensburg, Germany

**Keywords:** Multiple trauma patient, Thoracic trauma, Severely injured patient, TraumaRegister, Polytrauma, Major Trauma

## Abstract

**Background:**

Over the past, the severe thoracic trauma has had decisive influence on the outcome of multiple injured patients. Today, new therapies (e.g. extracorporeal membrane oxygenation (ECMO), protective ventilation methods and new forms of patient positioning) are available and applied regularly. What impact on the patient’s outcome does the thoracic trauma have today?

**Methods:**

Prospective data collection of multiple injured patients in a level-I trauma center was performed between 2008 and 2014. Patients with an ISS ≥16 were included and divided into 2 groups: Severe thoracic trauma (STT: AIS_Thorax_ ≥ 3) and mild thoracic trauma (MTT: AIS_Thorax_ < 3). In addition to preclinical and trauma room care, detailed information about clinical course and outcome were assessed.

**Results:**

In total, 529 patients (STT: *n* = 317; MTT: *n* = 212) met the in- and exclusion criteria. The mean Injury Severity Score (ISS) was significantly higher in patients of the STT group (STT: 33.5 vs. MTT: 24.7; *p* < 0.001), while the RISC II Score showed no significant differences (STT: 20.0 vs. MTT: 17.1; *p* = 0.241). Preclinical data revealed a higher intubation rate, more chest tube insertions and a higher use of catecholamines in the STT group (*p* < 0.05). Clinically, we found significant differences in the duration of invasive ventilation (STT: 7.3d vs. MTT: 5.4d; *p* = 0.001) and ICU stay (STT: 12.3d vs. MTT: 9.4d; *p* < 0.001). While the complication rate was higher for the STT group (sepsis (STT: 11.4% vs. MTT: 5.7%; *p* = 0.017); lung failure (STT: 23.7% vs. MTT: 12.3%; *p* = 0,001)), neither the non-adjusted lethality rate (STT: 13.2% vs. MTT: 13.7%; *p* = 0.493) nor the Standardized Mortality Ratio (SMR) showed significant differences (STT: 0.66 vs. MTT: 0.80; *p* = 0.397). The multivariate regressive analysis confirmed that severe thoracic trauma is not an independent risk factor for lethality in our patient cohort.

**Conclusion:**

Despite a higher injury severity, the extended need of emergency measures and a higher rate of complications in injured patients with severe blunt thoracic trauma, no influence on lethality can be proved. The reduction of the complication rate should be a goal for the next decades.

## Background

Severe multiple trauma is one of the tenth most common causes of death worldwide [1]. More than 50% of all severely injured patients (Injury severity score (ISS [2]) ≥ 16) suffer a relevant thoracic trauma [3]. In the past, thoracic trauma was associated with a high lethality rate. Severe thoracic injuries were considered to be responsible for 25% of all trauma deaths [4]. Furthermore, the lethality of multiple injured patients who sustained a thoracic trauma was assessed to be significantly higher than in multiple injured patients of equal severity without a thoracic trauma [5, 6].

These circumstances led to a continuous development and improvement of thoracic injury treatment. Nowadays, lung-protective, non-invasive ventilation protocols and early extubation are used to avoid ventilation induced lung injuries [7]. Compared to former controlled mechanical ventilation regimes, the early use of spontaneous breathing ventilation modalities is associated with a shorter duration of ventilatory support and a reduced length of intensive care unit (ICU) stay [8]. Moreover, these non-invasive ventilation modalities are helpful in case of an acute respiratory distress syndrome (ARDS) caused by a thoracic trauma [9]. In addition to these ventilation modalities the use of an extracorporeal membrane oxygenation (ECMO) in event of an ARDS can improve the outcome of severely injured patients, even in circumstances such as a hemorrhagic shock or coagulation failure [10].

Beside these treatment concepts, there are other medical achievements, which can improve the outcome of patients with a thoracic trauma, e.g. modern operative stabilization techniques of flail thoracic injuries [11] or the renaissance of prone positioning in ARDS [12].

Nevertheless, despite the described improvements, it is unclear whether these modern therapeutic concepts can improve the survival rate of multiple trauma patients with thoracic trauma injuries.

Therefore, the aim of the present study was to evaluate if there are still differences concerning the clinical outcome and lethality in multiple injured patients with or without a severe thoracic trauma.

## Methods

### Data collection

**Fig. 1:**
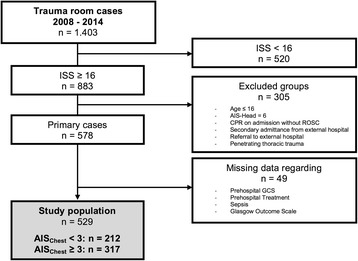
Selection of study sample

Over a period of 7 years (01.01.2008–31.12.2014) 1403 patients, treated in our level-I trauma center, were recorded in the Trauma Register DGU®, the trauma registry of the German Trauma Society (DGU) [13]. Of this collective, patients over 16 years and with an ISS ≥ 16 were included in this study. Patients were excluded if they were transferred from or to an outside hospital, in the event of unsuccessful emergency resuscitation and with an Abbreviated Injury Scale (AIS [14]) Head of 6. Additionally, all patients who sustained a penetrating thoracic trauma were excluded. In total, 578 patients met the inclusion criteria. The data collection of 49 patients was incomplete, therefore the study population consists of 529 patients (Fig. 1).

In contrast to other countries, the German trauma care system represents a decentralized system. In addition to different structural requirements, a level-I trauma center must fulfil at least 40 admissions with an ISS ≥16 [13, 15–17].

In total, about 500 parameters were collected prospectively in each single case during trauma room management by a study assistant. As well as the data of the TraumaRegister DGU® standard questionnaire (150 parameters, www.traumaregister-dgu.de), 350 other variables were collected to gain further information about the preclinical and clinical trauma care. The TraumaRegister DGU® standard questionnaire contains parameters concerning demography, individual treatment steps and relevant trauma scores: Glasgow Coma Scale (GCS) [18], Abbreviated Injury Scale (AIS) [14], Injury Severity Score (ISS) [2], New Injury Severity Score NISS [19], Glasgow Outcome Scale (GOS) [20] and Revised Injury Severity Classification II (RISC II), a prognostic tool for lethality in multiple injured patients [21]. In the present study, RISC II was used to calculate the expected lethality rate and to compute the standardized mortality ratio (SMR), which is defined as the quotient of the observed to the expected mortality.

### Ethical considerations

The study has been approved by the Institutional Review Board of the University of Regensburg (Number 14–101-0004). Data anonymity is guaranteed.

### Statistical analysis

A univariate data analysis was performed to compare patients with a severe thoracic trauma (AIS_Thorax_ ≥ 3, STT group, *n* = 317) and a mild thoracic trauma (AIS_Thorax_ < 3, MTT group, *n* = 212). The Chi-Square-Test (x^2^-Test) was used to analyze binary or nominal target variables. Mann-Whitney U-Test was performed in ordinal or metric values.

Two logistic regression analyses with the target variable “death in hospital” followed.

Firstly: An exploration of multiple dichotomous influence factors (age ≥ 60 yrs. vs. age < 60 yrs.; first measured blood pressure on-scene ≥90 mmHg vs. < 90 mmHg; Glasgow Coma Scale <9 vs. ≥ 9; AIS_Head_ ≥ 3 vs. < 3; AIS_Abdomen_ ≥ 3 vs. < 3; AIS_Extremities_ ≥ 3 vs. < 3; AIS_Thorax_ ≥ 3 vs. < 3; Volume prehospital & trauma room care ≥4000 ml vs. < 4000 ml; Organ failure; Multiple organ failure).

Secondly: Influence of severely injured body parts on lethality (AIS_Head_ ≥ 3 vs. < 3; AIS_Thorax_ ≥ 3 vs. < 3; AIS_Abdomen_ ≥ 3 vs. < 3; AIS_Extremities_ ≥ 3 vs. < 3).

All influence factors were included in a logistic regression analysis (backward elimination). *P*-values and odds ratios (OR) for each factor were calculated as well as the corresponding 95% confidence intervals (CI).

The statistical analysis (level of significance, *p* < 0.05) was carried out using SPSS software (SPSS Inc., Chicago, Illinois).

## Results

### Demographic characteristics

The STT group consists of 317 patients, the MTT group of 212 patients. The mean age was 44.8 ± 21.1 years; 73.3% of all included patients were male. No significant differences between the groups concerning age and sex could be identified. The mean ISS of all included patients was 30.0 ± 11.7, the mean NISS was 37.0 ± 15.0 with significant higher score values for the STT group compared to the MTT group (*p* < 0.001). The mean RISC II was 18.9 ± 27.8 with no significant differences between both groups. Different trauma mechanisms between the two groups could be identified. In the STT group we found a significantly higher rate of motor vehicle crashes as cause of injury, while low-height falls were more common in the MTT group (Table 1).

**Table 1:** Demographics, trauma scores and injury pattern

	Total *n* = 529	STT AIS Thorax ≥3 *n* = 317	MTT AIS Thorax <3 *n* = 212	*P*-value
Demographics and Trauma scores:				
- Male (n / %)	388 / 73.3	237 / 74.8	151 / 71.2	0.211
- Age (years ± SD)	44.8 (± 21.1)	43.9 (± 20.7)	46.1 (± 21.6)	0.260
- ISS (∅ ± SD)	30.0 (± 11.7)	33.5 (± 12.8)	24.7 (± 7.1)	<0.001
- NISS (∅ ± SD)	37.0 (± 15.0)	39.2 (± 14.9)	33.9 (± 14.7)	<0.001
- RISC II (∅ ± SD)	18.9 (± 27.8)	20.0 (± 29.0)	17.1 (± 25.8)	0.241
- Prehospital GCS (∅ ± SD)	10.6 (± 4.6)	10.5 (± 4.7)	10.7 (± 4.4)	0.817
Concomitant injuries:				
- AIS Head ≥3 (n / %)	304 / 57.5	153 / 48.3	151 / 71.2	<0.001
- AIS Abdomen ≥3 (n / %)	106 / 20.0	78 / 24.6	28 / 13.2	<0.001
- AIS Extremities ≥3 (n / %)	227 / 42.9	138 / 43.5	89 / 42.0	0.396
- AIS Extremities ≥2 (n / %)	384 / 72.6	246 / 77.6	138 / 65.1	0.001
- Number of diagnoses (∅ ± SD)	8.7 (± 4.0)	9.5 (± 4.0)	7.5 (± 3.7)	<0.001
- Number of surgeries (∅ ± SD)	5.4 (± 5.7)	5.8 (± 6.2)	4.7 (± 4.8)	0.021
Trauma mechanism:				
- Motor vehicle accident (n / %)	286 / 54.1	190 / 59.9	96 / 45.3	0.001
- Bicycle / Pedestrian (n / %)	67 / 12.7	37 / 11.7	30 / 14.2	0.239
- Falls <3 m (n / %)	64 / 12.1	24 / 7.6	40 / 18.9	<0.001
- Falls >3 m (n / %)	76 / 14.4	47 / 14.8	29 / 13.7	0.407
- Other (n / %)	36 / 6.8	19 / 6.0	17 / 8.0	0.231

As well as the trauma mechanism both groups showed significant differences concerning the injury pattern. The STT group showed more severe abdominal and limb injuries. In contrast to that, severe brain injuries were more common in the MTT group. Despite the higher incidence of severe brain injuries in the MTT group, the preclinical GCS showed no significant differences.

On average, the presence of a severe thoracic trauma (STT group) was associated with a higher number of clinical diagnoses and operations (Table 1).

### Preclinical care

Regarding the preclinical trauma sequence, intubation rate, rate of chest tube insertions and requirement of catecholamines were significantly higher in the STT group compared to the MTT group. Hemodynamic instability (RR syst. < 90 mmHg) occurred more often in patients with a severe thoracic trauma (STT group). While no significant differences in the preclinical rescue time could be identified between both groups, air-rescue was more common in the STT group (Table 2).

**Table 2:** Preclinical sequence and interventions

	Total *n* = 529	STT AIS Thorax ≥3 *n* = 317	MTT AIS Thorax <3 *n* = 212	*P*-value
Air-rescue (n / %)	350 / 66.2	221 / 69.7	129 / 60.8	0.022
Rescue time (min ± SD)	88.2 (±52.5)	86.5 (± 47.0)	90.7 (± 59.6)	0.964
Preclinical RR < 90 mmHg (n / %)	87 / 16.4	62 / 19.6	25 / 11.8	0.012
Preclinical intubation (n / %)	308 / 58.2	202 / 63.7	106 / 50.0	0.001
Preclinical chest tube (n / %)	71 / 13.4	67 / 21.1	4 / 1.9	<0.001
Preclinical CPR (n / %)	14 / 2.6	11 / 3.5	3 / 1.4	0.120
Preclinical catecholamine (n / %)	115 / 21.7	83 / 26.2	32 / 15.1	0.002

### Trauma room care

In patients of the STT group significantly lower hemoglobin values could be measured. However, there is no significant difference concerning massive transfusion of red blood cells (RBT ≥ 10 units of packed red blood cells) and circulatory instability, with only a slightly higher occurrence in the STT group. In 28.4% of all patients with a severe thoracic trauma (STT) chest tube insertion was performed during trauma room care. In order to substitute large volumes during the trauma room period, arterial lines and central venous accesses, e.g. CVC/Shaldon, were more frequently indicated in patients with a severe thoracic trauma (STT). In both groups, it took about 25 min until the whole-body-multislice-CT (WBMS-CT) was performed. No significant differences for the total trauma room time could be detected (Table 3).

**Table 3:** Interventions and data of trauma room care

	Total *n* = 529	STT AIS Thorax ≥3 *n* = 317	MTT AIS Thorax <3 *n* = 212	*P*-value
Trauma room intubation (n / %)	58 / 11.0	31 / 9.8	27 / 12.7	0.177
Trauma room chest tube (n / %)	99 / 18.7	90 / 28.4	9 / 4.2	<0.001
Trauma room CPR (n / %)	3 / 0.6	3 / 0.9	0 / 0.0	0.214
Trauma room catecholamine (n / %)	278 / 52.6	179 / 56.5	99 / 46.7	0.017
CVC / Shaldon (n / %)	186 / 35.2	128 / 40.4	58 / 27.4	0.001
Arterial line (n / %)	345 / 65.2	226 / 71.3	119 / 56.1	<0.001
RR <90 mmHg trauma room	74 / 14.0	51 / 16.1	23 / 10.8	0.056
Hemoglobin concentration g/dl (∅ ± SD)	12.2 (± 2.7)	11.9 (± 2.8)	12.6 (± 2.5)	0.004
Hemoglobin concentration < 9 g/dl (n / %)	63 / 12.0	42 / 13.6	20 / 9.5	0.100
RBT >10 units (n / %)	21 / 4.0	16 / 5.0	5 / 2.4	0.090
Whole-Body-Multislice-CT (WBMS-CT) (n / %)	506 / 95.7	309 / 97.5	197 / 92.9	0.011
Minutes until WBMS -CT (min ± SD)	25.3 (± 11.5)	25.9 (± 12.9)	24.2 (± 9.0)	0.312
Total length of trauma room care (min ± SD)	67.4 (± 34.5)	68.4 (± 33.9)	65.8 (± 35.5)	0.312

### Outcome

Significantly more complications such as sepsis, organ failure and respiratory failure occurred in the STT group. Only patients of the STT group were treated with an extracorporeal membrane oxygenation (ECMO) system. It should be noted, that the majority of ECMO-patients in our center got this treatment in peripheral hospitals by our air rescue ECMO-team and were then transferred. Because of this circumstance the majority of ECMO-patients were excluded. The duration of invasive ventilation and ICU stay, as well as the total hospital stay, was significantly longer in patients with a severe thoracic trauma (STT) compared to the MTT group (Table 4).

**Table 4:** Outcome indicators

	Total *n* = 529	STT AIS Thorax ≥3 *n* = 317	MTT AIS Thorax <3 *n* = 212	*P*-value
Organ failure (n / %)	344 / 65.0	221 / 69.7	123 / 58.0	0.004
Organ failure lungs (n / %)	101 / 19.1	75 / 23.7	26 / 12.3	0.001
ECMO (n / %)	9 / 1.7	9 / 2.8	0 / 0.0	0.010
Multi-organ failure (n / %)	207 / 39.1	133 / 42.0	74 / 34.9	0.062
Sepsis (n / %)	48 / 9.1	36 / 11.4	12 / 5.7	0.017
Thromboembolic event (n / %)	29 / 5.5	20 / 6.3	9 / 4.2	0.497
Length of intubation days (∅ ± SD)	6.6 (± 8.7)	7.3 (± 8.7)	5.4 (± 8.7)	0.001
Length of stay on ICU days (∅ ± SD)	11.1 (± 11.7)	12.3 (± 11.6)	9.4 (± 11.7)	<0.001
Length of stay in hospital days (∅ ± SD)	21.0 (± 15.4)	22.1 (± 16.0)	19.4 (± 14.3)	0.049
Mortality (n / %)	71 / 13.4	42 / 13.2	29 / 13.7	0.493
Standardized Mortality Ratio (SMR)	0.71	0.66	0.80	0.397
Glasgow Outcome Scale ≥4 (n / %)	368 / 69.6	224 / 70.7	144 / 67.9	0.289

The overall mortality rate was 13.4% without significant differences between patients with a severe (STT) and a mild thoracic trauma (MTT). Moreover, no significant differences concerning the standardized mortality ratio (SMR) could be detected. In both groups about 70% of the survivors showed a “good outcome” (Glasgow Outcome Scale ≥4) without significant differences.

### Logistic regression analysis

The first multivariate analysis showed that the severe thoracic trauma is not an independent predictor of lethality (*p* > 0.05). In contrast, massive transfusion of red blood cells (RBT ≥ 10 units of packed red blood cells) (OR 9.0; 95%-CI 2.6–31.3), an age over 60 years (OR 7.5; 95%-CI 4.0–14.0), GCS < 9 (OR 6.2; 95%-CI 3.4–11.4) and AIS_Extremities_ (OR 0.5; 95%-CI 0.2–0.9) were identified as independent risk factors.

The second multivariate regression analysis concerning the injury pattern showed that head injuries (AIS_Head_ ≥ 3) (OR 3.2; 95%-CI 1.7–5.9) and abdominal injuries (AIS_Abdomen_ ≥ 3) (OR 2.2; 95%-CI 1.2–4.1) were primary causes for death. The presence of a thoracic trauma had no influence on mortality.

## Discussion

This study deals with the influence of a severe thoracic trauma on the overall outcome and lethality rate of multiple injured patients.

In contrast to previously published investigations [5, 6] we could demonstrate for our population, that a severe thoracic trauma has no influence on the mortality rate and is no independent predictor for death in multiple injured patients.

Similar to previously published data [22–24], motor vehicle crashes could be identified as the most frequent injury mechanism for developing a severe thoracic trauma. Although, no significant difference of age between the two groups could be confirmed, low-height falls, as injury mechanism were much more common in the MTT group with a higher rate of traumatic brain injuries. Patients, who suffered a severe thoracic trauma (STT) showed a higher ISS compared to the other patients (MTT). Similar findings are published by Hill et al. [23].

Previous investigations showed a correlation between ISS and the mortality rate of multiple injured patients [25]. However, despite a higher ISS in the STT group, we could not find a higher mortality rate in these patients. This circumstance shows, that scoring systems that consider both, anatomic injury severity and physiologic parameters are potentially more qualified to predict lethality.

The preclinical time period in our collective was higher (88 min.) than the national average (69 min.) [3]. One reason could be the higher rate of air rescue transports (66.2% vs. national average: 40.2%) [3]. Nevertheless, Kleber et al. showed that the preclinical time period has no influence on the overall lethality rate of multiple injured patients [26].

Especially patients with a severe thoracic trauma showed a higher rate of preclinical procedures (e.g. intubation: 63.7%; chest tube insertion: 21.1%) compared to the national average [3] or previously published data [27]. Bayer et al. also observed a higher rate of preclinical procedures in patients with a severe thoracic trauma compared to patients without (intubation: 44% vs. 36.3%; chest tube insertion: 11% vs. 1.9%) [22]. However, compared to the data of Bayer et al., the preclinical procedure rate was higher in our collective. This could be explained by a higher injury severity of our patient cohort (mean ISS: 30.0 vs. 25.6) or a higher rate of preclinical procedures in air rescue transport, demonstrated by Andruszkow et al. [28].

Patients who suffered a severe thoracic trauma showed a significantly extended period of invasive respiration (mean 7 days) and length of ICU stay (mean 12 days) compared to patients with a mild thoracic trauma. These findings are comparable with previously published data (invasive respiration: 7d; ICU: 11d [29]; invasive respiration: 8d; ICU: 11d [27]). Additionally, the complication rate (e.g. organ failure, respiratory failure, sepsis) was higher in the STT group compared to the MTT group. Whereas Trupka et al. reported that patients with a thoracic trauma are significantly more likely to develop multiple organ failure [30], we could not find differences between both groups.

Over the last decades a decline of lethality in trauma patients could be observed [31, 32]. Previous studies presented considerably higher lethality rates in patients who suffered a thoracic trauma (7.6% (ISS = 16.4) [33]; 18.7% (ISS = 26.7) [34]).

In contrast to prior studies [5, 6] our study shows no further influence of thoracic trauma to lethality in multiple injured patients. The lethality in our study (13.2% (STT) vs. 13.7% (MTT)) is lower than the lethality rates of other German multicenter studies (Timm et al. 17%, SMR 0.82 [3], Huber et al. 17.5% [29]). This could be due to the high case load of our trauma center (>100/year). Zacher et al. [35] come to the conclusion that the hospital volume is as an independent predictor of survival. A case load of 40 patients per year per hospital appeared beneficial for survival.

A direct international comparison of trauma centers has not yet been described in the literature. Nevertheless, similar mortality rates have been published for comparable patient populations in the USA and the Netherlands [36].

In our opinion, the approximation of the lethality rates in both groups is above all due to advances in intensive care treatment for patients with thoracic trauma over the past years: Nowadays, lung-protective, non-invasive ventilation protocols and early extubation are used to avoid ventilation induced lung injuries [7]. The early use of spontaneous breathing ventilation modalities is associated with a shorter duration of ventilatory support and a reduced length of intensive care unit (ICU) stay [8]. Moreover, non-invasive ventilation modalities reduce reintubation rates and are useful in case of an acute respiratory distress syndrome (ARDS) caused by a thoracic trauma [9]. The use of an extracorporeal membrane oxygenation (ECMO) can improve the treatment and outcome of severely injured patients, including circumstances such as hemorrhagic shock [10]. The ECMO also achieves a considerable improvement of paO_2_/FiO_2_-ratio, pH-levels and pCO_2_ [37]. As well as these treatment concepts, there are other medical achievements, which can improve the outcome of patients with a thoracic trauma, e.g. modern operative stabilization techniques of flail thoracic injuries [11] or the renaissance of prone positioning in ARDS [12].

In both groups, less patients died than expected by RISC II, the SMR was 0.66 in the severe thoracic trauma and 0.80 in the mild thoracic trauma group without significance difference. This finding was unexpected, especially the considerably lower SMR in the STT group. An explanation for this could be the significantly higher number of severe head injuries and the slightly older population in the MTT Group. Further studies on this topic will be necessary.

The severe thoracic trauma was not an independent predictor for lethality in the multivariable analysis. Similarly to previously published data, in our collective massive transfusion, an age over 60 years and GCS < 9 were found to be independent risk factors for lethality [24, 38, 39].

Also, the second multivariable analysis – based on injury pattern – does not unmask thoracic trauma as a risk for death. Brain and abdominal injuries could be detected to be responsible for lethality.

Through comparing our results with previously published data we can assume that modern treatment strategies (e.g. lung protective ventilation; prone positioning; ECMO) could have a positive influence on the mortality rate and the overall outcome of multiple injured patients with a severe thoracic trauma.

### Limitations

Despite a big and precisely defined patient collective some limitations of the present study should be noted: Firstly, despite the prospective data collection, it is a register study with the known-weaknesses. Secondly, it is a single-center-study without the possibility to transfer the results to a general population. However, we do assume, that our therapy standards for preclinical, trauma room and ICU treatment (this includes ATLS/S3 guidelines) are comparable to other level-I trauma centers in Germany/high-income countries.

Further studies are needed to evaluate the influence of different treatment methods on the outcome of multiple injured patients with a severe thoracic trauma.

## Conclusions

In contrast to prior studies, we show that in our study population the severe thoracic trauma has no direct influence on lethality in multiple injured patients. Patients who suffered a severe thoracic trauma still present a higher injury severity, an extended need of preclinical procedures and a higher rate of complications, but no difference in lethality in comparison to patients with a mild thoracic trauma. Further reduction of the lethality rate and a reduction of the complication rate should be a goal for the next decades.

## Abbreviations

∅: Mean value
AIS: Abbreviated Injury Scale
ARDS: Acute respiratory distress syndrome
CI: Confidence interval
CO_2_: Carbon dioxide
CT: Computer tomography
CVC: Central venous catheter
DGU: Deutsche Gesellschaft für Unfallchirurgie e.V.
ECMO: Extracorporeal membrane oxygenation
GCS: Glasgow Coma Scale
GOS: Glasgow Outcome Score
Hb: Hemoglobin
ICU: intensive care unit
ISS: Injury Severity Score
MTT: Mild thoracic trauma
NISS: New Injury Severity Score
OR: odds ratio
paO_2_/FiO_2_: Partial pressure arterial oxygen / fraction of inspired oxygen
PTT: Partial thromboplastin time
RBT: Red blood cells transfusion
RISC II: Revised Injury Severity Classification II
RR: Blood pressure
SMR: Standardized mortality rate
STT: Severe thoracic trauma
WBMS-CT: Whole-Body-Multislice-CT
